# Diacylglycerol kinase (DGKA) regulates the effect of the epilepsy and bipolar disorder treatment valproic acid in *Dictyostelium discoideum*

**DOI:** 10.1242/dmm.035600

**Published:** 2018-08-21

**Authors:** Elizabeth Kelly, Devdutt Sharma, Christopher J. Wilkinson, Robin S. B. Williams

**Affiliations:** Centre for Biomedical Sciences, School of Biological Sciences, Royal Holloway University of London, Egham TW20 0EX, UK

**Keywords:** Diacyclglycerol, Diacylglycerol kinase, *Dictyostelium discoideum*, Epilepsy, Lithium, Valproic acid

## Abstract

Valproic acid (VPA) provides a common treatment for both epilepsy and bipolar disorder; however, common cellular mechanisms relating to both disorders have yet to be proposed. Here, we explore the possibility of a diacylglycerol kinase (DGK) playing a role in regulating the effect of VPA relating to the treatment of both disorders, using the biomedical model *Dictyostelium discoideum*. DGK enzymes provide the first step in the phosphoinositide recycling pathway, implicated in seizure activity. They also regulate levels of diacylglycerol (DAG), thereby regulating the protein kinase C (PKC) activity that is linked to bipolar disorder-related signalling. Here, we show that ablation of the single *Dictyostelium dgkA* gene results in reduced sensitivity to the acute effects of VPA on cell behaviour. Loss of *dgkA* also provides reduced sensitivity to VPA in extended exposure during development. To differentiate a potential role for this DGKA-dependent mechanism in epilepsy and bipolar disorder treatment, we further show that the *dgkA* null mutant is resistant to the developmental effects of a range of structurally distinct branched medium-chain fatty acids with seizure control activity and to the bipolar disorder treatment lithium. Finally, we show that VPA, lithium and novel epilepsy treatments function through DAG regulation, and the presence of DGKA is necessary for compound-specific increases in DAG levels following treatment. Thus, these experiments suggest that, in *Dictyostelium*, loss of DGKA attenuates a common cellular effect of VPA relating to both epilepsy and bipolar disorder treatments, and that a range of new compounds with this effect should be investigated as alternative therapeutic agents.

This article has an associated First Person interview with the first author of the paper.

## Abstract (precis)

**Editor's choice:** Here, using a tractable model system, *Dictyostelium discoideum*, we show that diacylglycerol kinase activity might contribute to the cellular mechanism of action of the epilepsy and bipolar disorder treatment, valproic acid.

## INTRODUCTION

Diacylglycerol kinases (DGKs) provide the first step in the phosphoinositide salvage pathway, functioning in the phosphorylation of diacylglycerol (DAG) to produce phosphatidic acid (PA) (Whatmore et al., 1999). Through this activity, cells regulate levels of lipid (Milne et al., 2008) and glucose metabolism (Banfic et al., 1993; Inoguchi et al., 1994), cell growth (Banfic et al., 1993) and cell signalling (Cerbón et al., 2005). In addition, DGKs regulate both inositol phosphate and phosphatidylinositol signalling through the recycling of DAG, and activation of protein kinase C (PKC; also known as PRKC) enzymes, which require DAG binding. Owing to the numerous roles of DAG, and the ten DGK isoforms in humans, few studies have considered a role for DGK-dependent signalling in disease states. The exceptions to this are several knockout studies that have linked various DGK isoforms with epilepsy [DGKβ (Ishisaka et al., 2103), DGKδ (Leach et al., 2007) and DGKε (Rodriguez de Turco et al., 2001)] and bipolar disorder [DGKβ (Kakefuda et al., 2010; Squassina et al., 2009) and DGKη (Baum et al., 2007; Moya et al., 2010)]. In bipolar disorder, PKC levels are also elevated during manic episodes (Friedman et al., 1993; Wang and Friedman, 1996), and lithium, a common bipolar disorder treatment, is known to increase DAG levels (Brami et al., 1993; Drummond and Raeburn, 1984). Surprisingly, no studies, to our knowledge, have investigated DGK-dependent signalling as an overlapping process relating to both epilepsy and bipolar disorder treatment.

One widely used treatment for both epilepsy and bipolar disorder is the eight-carbon, branched-chain fatty acid, valproic acid (VPA). It was discovered accidentally in 1963 as a treatment for epilepsy (Meunier et al., 1963), and is also used in the treatment of migraine (Pryse-Phillips et al., 1997), with potential for the treatment of cancer (Arce et al., 2006) and HIV (Lehrman et al., 2005). In a clinical context, it is used at a plasma concentration of 0.3-0.6 mM (DSMIV, 2000), but it is teratogenic, leading to enhanced likelihood of birth defects if taken during pregnancy, thus limiting its clinical use (Jentink et al., 2010). To develop novel compounds lacking the side effects of VPA, but with a common therapeutic mechanism, many studies have sought to identify its cellular effects and potential targets. This research has been complicated by a broad range of effects caused by VPA, linked to varying therapeutic (or adverse) mechanisms (Terbach and Williams, 2009). One approach to simplify this research is to use a tractable model system, such as the simple biomedical model *Dictyostelium discoideum.*

*Dictyostelium* has been used to investigate complex cellular mechanisms of both bioactive natural products and drugs, including VPA. This pharmacogenetics research has employed *Dictyostelium*, because its haploid genome enables the rapid identification and ablation of genes encoding proteins that control sensitivity to a drug in growth or acute cell behaviour responses, thus implicating either the identified protein or the pathway that it regulates in the action of a drug. In such an approach, the mutant cell line might also show reduced sensitivity to the effects of the compound on multicellular development, thus conferring resistance to the drug-dependent block in fruiting body formation. *Dictyostelium* provides an excellent system to investigate the developmental effects of VPA, because its well-defined process of cell migration, coalescence and differentiation to form multicellular fruiting bodies is sensitive to VPA (Boeckeler et al., 2006; Williams et al., 2002). It is important to note that *Dictyostelium* provides the ability to assess these behaviours as mutually exclusive cell functions, controlled by groups of both independent and common proteins, yielding a considerable advantage over other model systems. *Dictyostelium* was the first model to show VPA-dependent effects on phospholipid signalling (Chang et al., 2012; Xu et al., 2007) relating to seizure control (Chang et al., 2013, 2014, 2015, 2016), and on inositol phosphate signalling (Eickholt et al., 2005; Frej et al., 2016; Shimshoni et al., 2007; Williams et al., 2002) relating to bipolar disorder treatment. These studies have shown that VPA reduces phosphoinositide levels in a time- and dose-dependent manner (Chang et al., 2012; Xu et al., 2007), independent of phosphatidylinositol-3-kinase activity, inositol recycling and *de novo* inositol biosynthesis. In *Dictyostelium*, VPA has also been found to increase the levels of phospholipids, phosphatidylcholine (PC) and phosphatidylethanolamine (PE) (Elphick et al., 2012), implicating the phosphoinositide salvage pathway as a target pathway of the drug. In both epilepsy and bipolar disorder, *Dictyostelium* has been used to identify mechanisms underlying the therapeutic role of VPA and also novel compounds for seizure control that have been validated by confirmatory experiments in both *in vitro* and *in vivo* mammalian models (Chang et al., 2012, 2014, 2015; Williams et al., 2002). These various studies suggest that *Dictyostelium* could provide valuable insights into the mechanism of VPA in therapeutic function, relating to both epilepsy and bipolar disorder treatment.

Here, we investigate a role for DGKA in attenuating the effect of VPA in *Dictyostelium*. The use of *Dictyostelium* as a model provides the opportunity to ablate the single *dgkA* gene, creating a stable isogenic cell line lacking all DGK activity, and enabling the subsequent quantification of acute effects of VPA and congeners on cell behaviour and development in the absence of this enzyme (Chang et al., 2012; Cocorocchio et al., 2016, 2018; Xu et al., 2007). Previous studies show that ablation of the *dgkA* gene gives rise to altered development, where cells were able to form small, but relatively normal, fruiting bodies (Egelhoff et al., 1993), but this study was complicated by the proposal that the enzyme functioned as a myosin II kinase, whereas it was later shown to provide DGK activity (De La Roche et al., 2002; Ostroski et al., 2005). We show that loss of DGKA, a proposed DGKθ orthologue, results in a significant decrease in the potency of VPA in triggering acute cell behaviour responses and in development. We further show that loss of DGKA reduces sensitivity to the inhibitory effects of a range of other potential epilepsy treatments and a structurally dissimilar bipolar disorder treatment, lithium. These results suggest that DGKA might regulate the cellular effects of VPA, relating to treatments for both epilepsy and bipolar disorder, and newly identified alternatives to VPA could function through the same molecular mechanism.

## RESULTS

### *Dictyostelium* DGKA represents the origins of the family of mammalian DGK enzymes

**Fig. 1.**
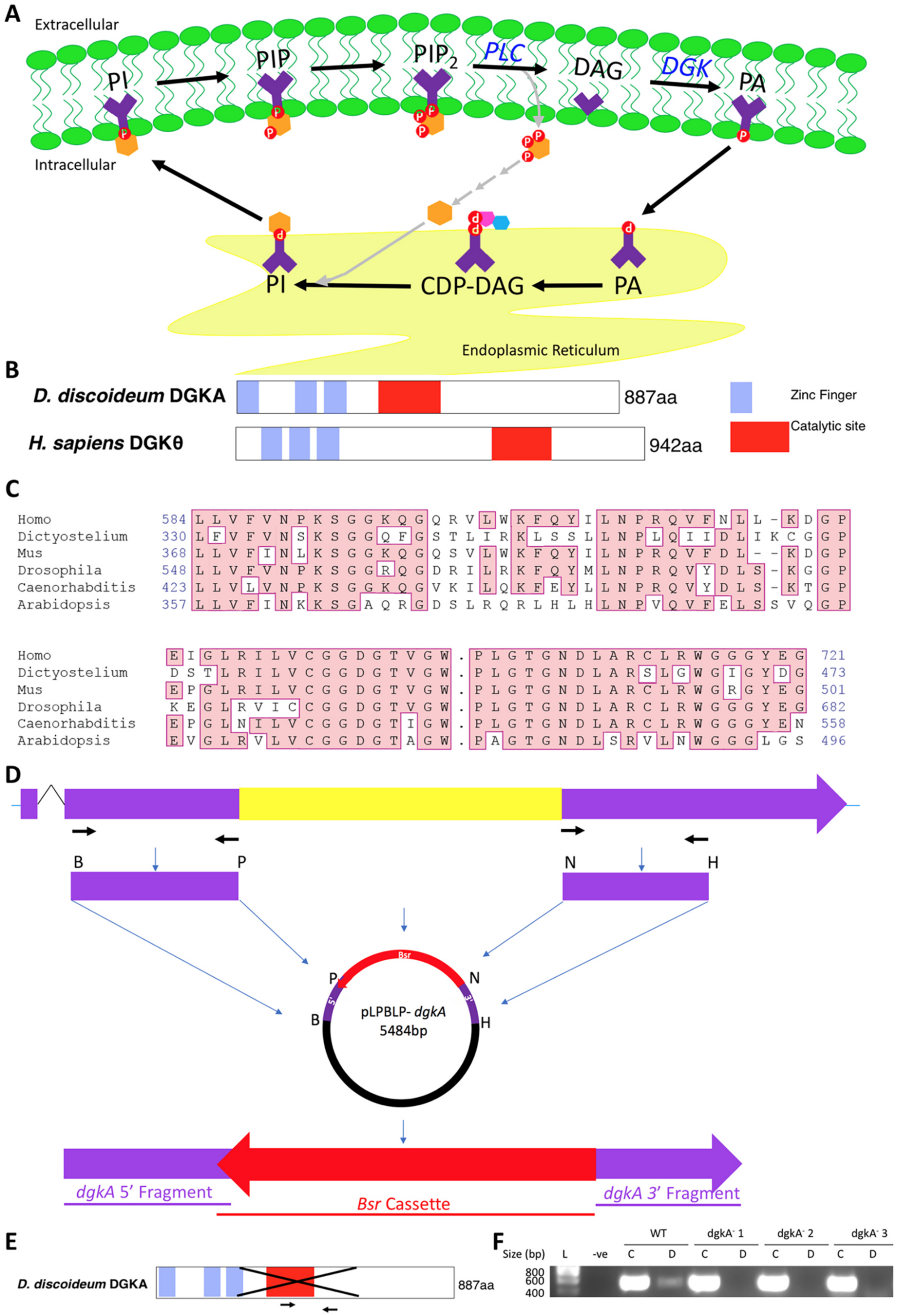
**Diacylglycerol kinase (DGK), within the phosphoinositide salvage pathway, is highly conserved across kingdoms and ablated in *Dictyostelium*.** (A) Schematic of the phosphoinositide salvage pathway, showing the recycling of diacylglycerol (DAG) to phosphatidic acid (PA) from the extracellular membrane to the endoplasmic reticulum, via cytidine diphosphate (CDP)-DAG to phosphoinositide (PI), and then back to the extracellular membrane and phosphorylation to various phosphoinositides (PIP, PIP_2_). Phospholipase C (PLC) regenerates DAG and inositol trisphosphate. (B) Schematic representation of *Dictyostelium* DGKA and human DGKθ, highlighting DAG binding domains and the catalytic domain. (C) Conservation amino acids within regions of the DGK catalytic domain (pink), with ‘**.**’ representing an intervening region. (D) Schematic of homologous recombination used to produce the *dgkA* knockout fragment, with arrows representing the primers used to create the two fragments which were inserted into the knockout vector, and the area in yellow indicating the region deleted. (E,F) A DGKA null mutant was created, with the deletion of the core catalytic domain of the protein (E). Arrows represent primers used to demonstrate loss of expression (RT-PCR) in the mutant (F).

Because DGKs catalyse the first step in the DAG salvage pathway (Fig. 1A), we initially investigated homology between the single *Dictyostelium* DGKA protein and the ten members of the mammalian DGK family of enzymes (Fig. 1B-F). The *Dictyostelium* DGKA protein shows a conserved domain structure generally found in the ten human isoforms (Fig. S1), with three putative N-terminal phorbol-ester/DAG-type 1 zinc finger domains and a DAG-kinase catalytic domain (Fig. 1B). The catalytic site is highly conserved between the human and *Dictyostelium* proteins and broadly conserved throughout other kingdoms (Fig. 1C). In addition, the *Dictyostelium* enzyme retains a conserved glycine (G262) that, when mutated, abolishes enzymatic activity in COS-7 cells without effect on translocation to membrane (Los et al., 2004; van Baal et al., 2005) and two prolines (P245 and P246) necessary for full enzymatic activity (Los et al., 2004). The *Dictyostelium* protein also contains three cysteine residues necessary for membrane association of the mammalian protein (van Baal et al., 2005). Although the *Dictyostelium* protein shows slightly stronger homology to the human DGKθ (De La Roche et al., 2002), the enzyme is likely to function as a single, generic DGK owing to high sequence conservation between *Dictyostelium* DGKA and all ten human isoforms (Fig. S2). The cladistics tree supports evolutionary conservation of the DGKs with tight clusters of DGK types 1-5 in organisms with multiple isoforms (Fig. S3).

To analyse the role of the *Dictyostelium* DGKA enzyme, we ablated the enzyme in an isogenic cell line. The *dgkA^−^* mutant was produced by homologous recombination with a knockout cassette, leading to the loss of 1072 bp from the central region of the encoding gene including the catalytic site (Fig. 1F; Fig. S4). Loss of *dgkA* gene expression was confirmed by reverse transcription polymerase chain reaction (RT-PCR) (Fig. 1E). The *dgkA^−^* mutant was then used to investigate sensitivity or resistance to VPA exposure in both acute cell behaviour assays and in development.

### Investigating a role for DGKA in regulating the acute effect of VPA on *Dictyostelium* cell behaviour

**Fig. 2.**
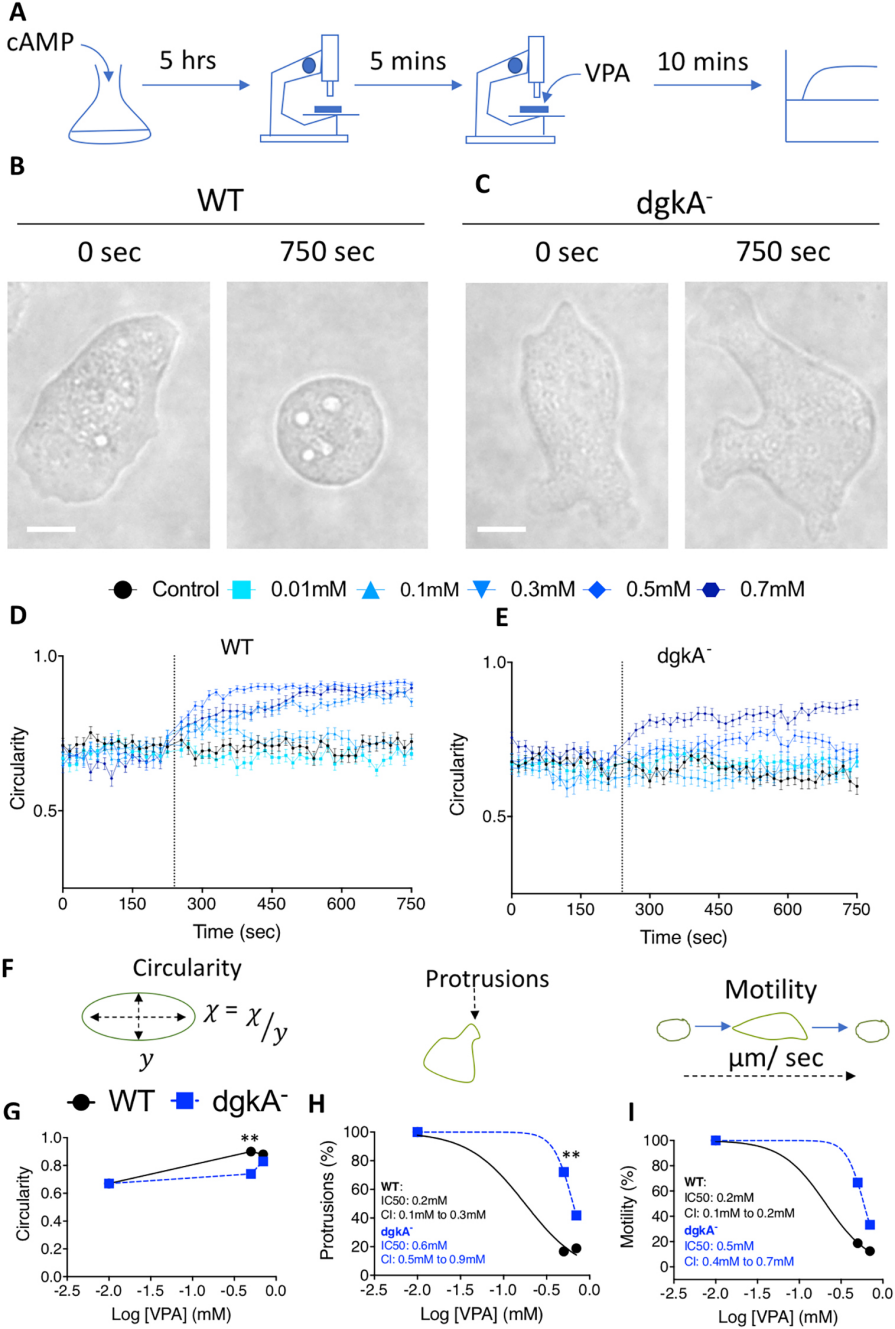
**Loss of DGKA confers reduced sensitivity to the acute effects of VPA.** (A) Schematic of the acute cell behaviour experimental procedure. Cells were induced to early development by pulsatile cAMP before being visualised under a microscope, and cell movement was recorded in the absence and presence of VPA for 250 s and 750 s, respectively. (B,C) Brightfield images before and after addition of 0.5 mM VPA to WT (B) and *dgkA^−^* cells (C). Scale bars: 4 µm. (D,E) Quantification of cell circularity in the absence and presence of a range of VPA concentrations (0.01 mM to 0.7 mM) in WT (D) and *dgkA^−^* (E) cells, with data presented as mean±s.e.m. (*n*=30 cells). (F-I) Schematic of the quantitative measurements taken during the assay (F). Black dashed line arrows represent the measurement being taken and the resulting secondary plots for data normalised to control conditions of each cell type (100%) for circularity (G), membrane protrusions (H) and motility (I) for WT (black circles) and *dgkA^−^* (blue squares) cells. A Kruskal–Wallis with Dunn’s post hoc test was used to compare WT and *dgkA^−^* cell lines. There were significant differences in WT and *dgkA^−^* cell displacement, circularity, protrusions and motility, as indicated. IC_50_, half maximal inhibitory concentration; CI, 95% confidence interval. **P≤0*.*01*.*

Because we have previously shown that VPA acutely blocks *Dictyostelium* cell behaviour (Boeckeler et al., 2006; Williams et al., 2002), we then assessed whether this effect was attenuated following ablation of DGKA. This was carried out by monitoring cell behaviour in wild-type and *dgkA^−^* cells prior to treatment, to identify potential behavioural differences owing to loss of DGKA, and following exposure to VPA, to identify altered sensitivity to the behavioural changes caused by VPA. The logic to this approach is that loss of VPA sensitivity in the mutant would implicate a role for DGKA-related signalling or protein function in the effect of VPA on acute cell behaviour in this model. In these experiments, cells were treated with pulsatile cyclic adenosine monophosphate (cAMP) to mimic the natural signalling pathways *Dictyostelium* uses to initiate the process of changing from a unicellular lifestyle to a multicellular phase, beginning with aggregation. These cells, in early development with rapid cell movement, were then exposed to VPA for 10 min to monitor changes in cell behaviour (Fig. 2A). As an initial approach, we treated cells with VPA at 0.5 mM to show that wild-type cells had halted in movement and formed a circular shape (Fig. 2B), whereas *dgkA^−^* cells continued to move and remained amoeboid in shape (Fig. 2C), after VPA treatment. We continued this analysis by employing a recently developed assay for monitoring acute changes in cell behaviour following drug or compound exposure, enabling quantification of cell responses (Cocorocchio et al., 2016, 2018). Here, cell behaviour was recorded using time-lapse microscopy prior to and following the addition of VPA, and computer-generated cell outlines were analysed to describe changes in cell shape (circularity) (Fig. 2D-F), membrane protrusion (Fig. 2G) and motility (Fig. 2H). In the absence of VPA treatment, wild-type and *dgkA*^–^ cells showed similar circularity (0.71±0.02 and 0.65±0.04, respectively), protrusions (8.4±0.1 and 9.2±0.1, respectively) and motility (0.012±0.001 and 0.017±0.001 µm/s, respectively, suggesting that these characteristics and behaviours are not grossly affected by loss of DGKA. Following treatment, in all cell behaviour characteristics monitored, *dgkA^−^* cells showed reduced sensitivity to the acute effects of VPA, with around a twofold reduction in IC_50_ values relating to membrane protrusions and motility compared with those of wild-type cells (Fig. 2G,H). This was also seen when comparing the average protrusion number in the absence and presence of VPA (0.5 mM), with wild-type cells showing a 5.6-fold reduction, and *dgkA^−^* cells showing a 1.5-fold reduction, following treatment. These results suggest that, following the deletion of *dgkA*, the effect of VPA on acute cell behaviour is reduced, implicating either a role for DGKA-dependent signalling or the DGKA protein in regulating the action of VPA in this model.

### Rescuing the DGKA-dependent VPA sensitivity in acute cell behaviour

**Fig. 3.**
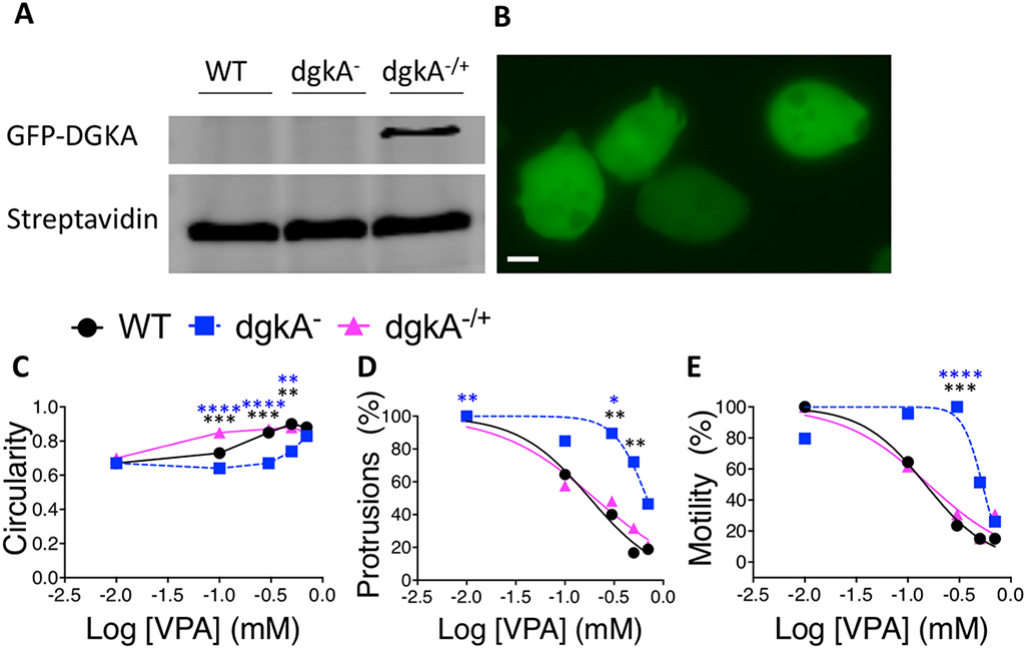
**VPA sensitivity is restored by expression of GFP-DGKA in the *dgkA* null mutant.** (A) Western blot showing GFP-DGKA expression with loading control (streptavidin) for WT, *dgkA^−^* and *dgkA^−/+^* cell lines. (B) Fluorescence image of GFP-DGKA localisation in *dgkA^−/+^* cells. Scale bar: 10 µm. (C-E) In behavioural assays, cells were induced to early development by pulsatile cAMP, before cell behaviour was recorded in the absence and presence of VPA for WT (black circles), *dgkA^−^* (blue squares) and *dgkA^−/+^* (pink triangles) cell response to 0.01-0.7 mM VPA for circularity (C), protrusions (D) and motility (E). Data are from *n*=30 cells, with secondary plots using data normalised to control conditions of each cell type (100%). A Kruskal–Wallis with Dunn’s post hoc test was used to compare the three cell lines, for which there were significant differences between WT and *dgkA^−^* (black asterisks), and *dgkA*^−^ and *dgkA*^−/+^ (blue asterisks), cells as indicated. *P≤0*.*05, **P≤0*.*01, ***P<0*.*001 and ****P≤0*.*0001*.*

We next investigated whether the reduced VPA sensitivity in acute cell behaviour shown for *dgkA^−^* cells was through loss of DGKA, by expression of a tagged DGKA-GFP in *dgkA^−^* cells (to produce *dgkA^−/+^*). In these experiments, the expressed DGKA-GFP protein was of the expected size (130 kDa, Fig. 3A), and was localised within the cytosol (Fig. 3B), consistent with the localisation of the mammalian protein (van Baal et al., 2005). Acute cell behaviour analysis of the resulting *dgkA^−/+^* cells showed reinstated VPA sensitivity in relation to circularity, protrusion formation and motility (Fig. 3C-E; Fig. S5). These results further support a role for DGKA-dependent signalling or the DGKA protein in regulating the sensitivity of cells to the effects of VPA.

### Investigating a role for DGKA in regulating the effect of VPA on *Dictyostelium* development

**Fig. 4.**
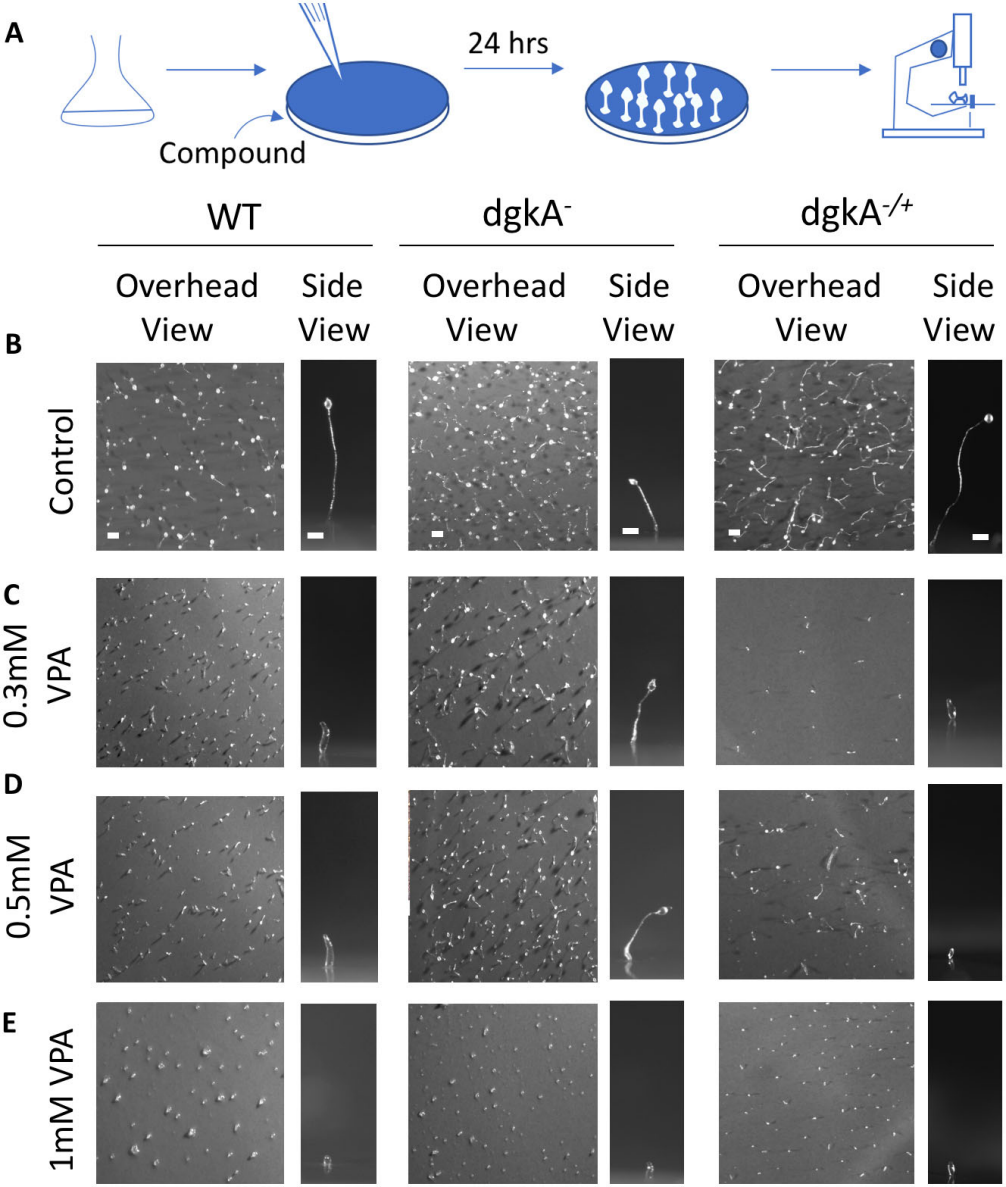
**Loss of DGKA confers reduced sensitivity to the chronic effects of VPA on development.** (A) Schematic of the development assay experimental procedure. Cells were shaken and placed on a nitrocellulose filter in the presence and absence of VPA for 24 h to enable development before being visualised under a dissection microscope. (B-E) Overhead and side shots of developmental morphologies (B) under control conditions, and in the presence of VPA at (C) 0.3 mM, (D) 0.5 mM and (E) 1 mM, in WT (left column), *dgkA*^−^ (middle column) and *dgkA*^−/+^ (right column) cells. Data are representative of triplicate independent experiments. Scale bars: 0.5 mm (overhead view) and 0.1 mm (side view).

In *Dictyostelium*, VPA has been shown to block development, where starving cells aggregate to form a mound and then develop into a mature fruiting body over a 24 h period (Boeckeler et al., 2006; Williams et al., 2002) (Fig. 4A). Because the development of *dgkA^−^* cells produces morphologically normal fruiting bodies, with distinct stalk and spore heads (Egelhoff et al., 1993), albeit of reduced size, we can therefore analyse the sensitivity of this mutant to the VPA-dependent block in fruiting body formation. Loss of VPA sensitivity in the mutant would suggest that VPA functions to block development in *Dictyostelium* through a mechanism dependent upon DGKA-related signalling or protein function. We therefore compared fruiting body morphology, in wild-type, *dgkA^−^* and *dgkA^−/+^* cells, starved on nitrocellulose filters (Fig. 4B-E), in the absence and presence of a range of VPA concentrations (0.3 mM, 0.5 mM and 1 mM) (Fig. 4C-E). The therapeutic (plasma) concentrations of VPA in patients is ∼0.4 mM (DSMIV, 2000). In the absence of VPA, wild-type, *dgkA^−^* and *dgkA^−/+^* cells formed mature fruiting bodies, containing a stalk and a spore head, although the overall size of the *dgkA^−^* fruiting body was reduced compared with that of wild-type cells, consistent with previous reports (Abu-Elneel et al., 1996; De La Roche et al., 2002) (Fig. 4B). Fruiting body size was restored in the *dgkA^−/+^* rescue cell line compared with that of wild-type cells, consistent with complementing the loss of *dgkA* (Fig. 4B). In the presence of VPA at 0.3 mM (Fig. 4C) and 0.5 mM (Fig. 4D), wild-type fruiting body morphology was severely affected, reducing the aggregate size and stopping development at the mound stage at 1 mM (Fig. 4E), consistent with earlier studies (Boeckeler et al., 2006; Williams et al., 2002). In contrast, *dgkA^−^* cells were able to develop into mature fruiting bodies at 0.3 mM (Fig. 4C) and 0.5 mM (Fig. 4D) that were indistinguishable from those formed in the absence of VPA, although development was also arrested at the mound stage in the presence of 1 mM VPA (Fig. 4E). VPA sensitivity was restored in the *dgkA^−/+^* cell line; fruiting body development was halted with a similar effect to that shown for wild-type cells (Fig. 4C,D). These results suggest that the developmental effect of VPA on *Dictyostelium* is at least partially dependent upon the presence of the DGKA protein.

### Investigating a role for DGKA in regulating the effect of other epilepsy and bipolar disorder treatments on *Dictyostelium* development

**Fig. 5.**
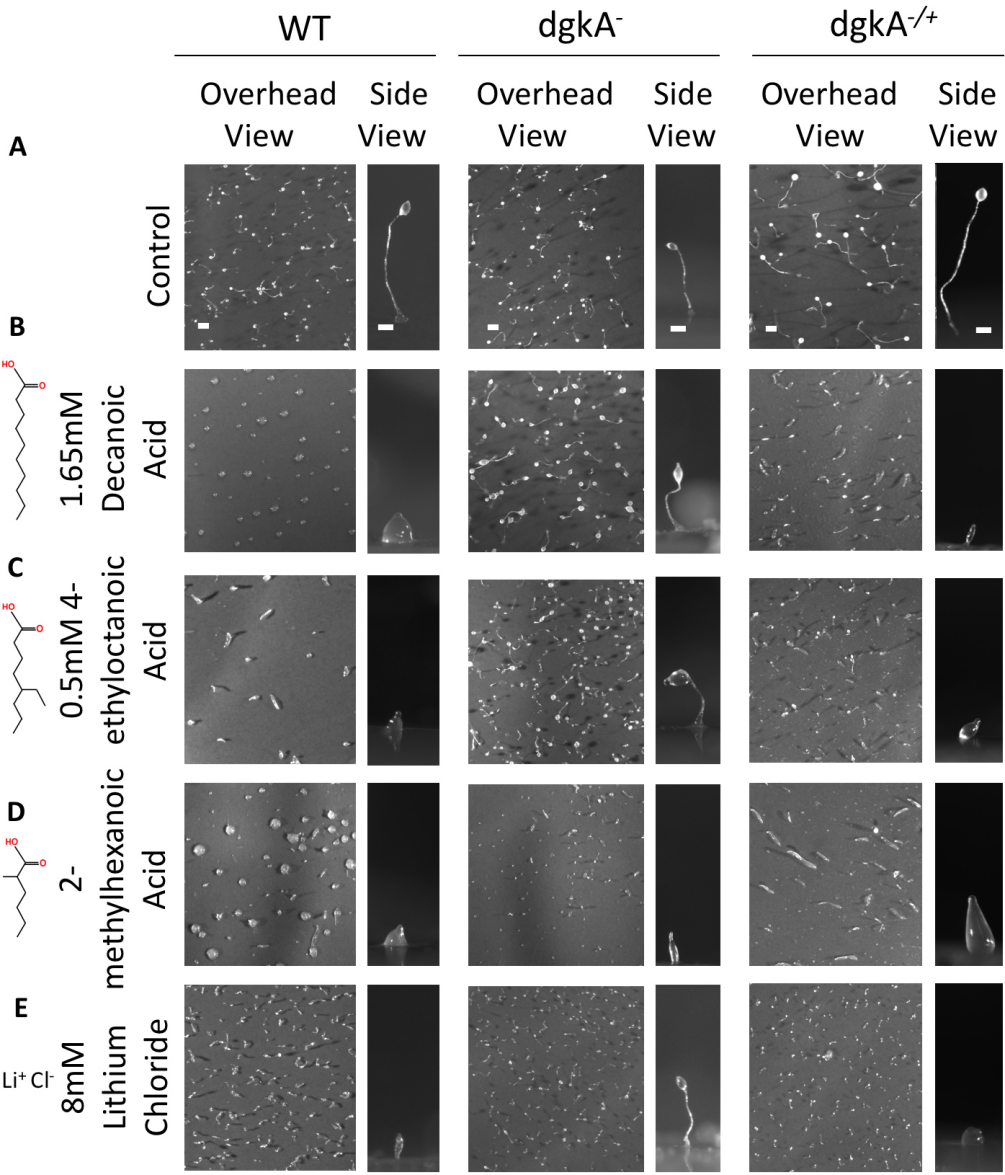
**Loss of DGKA confers** **reduced sensitivity to the chronic developmental effects of a range of novel antiepileptic treatments and the bipolar disorder treatment, lithium chloride.** Overhead and side view images of development in WT (left column), *dgkA*^−^ (middle column) and *dgkA*^−/+^ (right column) cells (A) under control conditions and in the presence of (B) decanoic acid (1.65 mM), (C) 4-ethyloctanoic acid (0.5 mM), (D) 2-methylhexanoic acid (0.5 mM) and (E) lithium chloride (8 mM). Data are representative of triplicate independent experiments. Scale bars: 0.5 mm (overhead view) and 0.1 mm (side view).

Research into the molecular mechanisms of VPA has yet to identify a common mechanism for both epilepsy and bipolar disorder treatments. We therefore investigated the role of the *Dictyostelium* DGKA protein in regulating the effect of VPA relating to the treatment of both epilepsy and bipolar disorders, using a range of epilepsy treatments shown to be active in *Dictyostelium* and in mammalian epilepsy models (Chang et al., 2012, 2013, 2014, 2015, 2016) and the commonly used bipolar disorder treatment, lithium (Williams et al., 1999, 2002). The logic to this approach was that if these compounds modulate the same signalling pathway or have the same protein target, cells in which this pathway is blocked or has a missing target will show reduced responsiveness to each compound. This analysis was limited to assessing the developmental effects of these compounds, because lithium does not show acute effects on cell behaviour in *Dictyostelium* (King et al., 2009). In these experiments, cells were again starved on nitrocellulose filter for 24 h to initiate development, in the presence of a range of compounds at concentrations that inhibited development and fruiting body formation in wild-type cells, and the effects were compared between wild-type and *dgkA^−^* cells (Fig. 5A). We first investigated decanoic acid, a compound that attenuated phosphoinositide signalling in *Dictyostelium* and provides a key component of medium-chain ketogenic diet for the treatment of drug-resistant epilepsy, with efficacy against seizure activity in an *ex vivo* hippocampal slice model (Augustin et al., 2018; Chang et al., 2016). This compound arrested wild-type *Dictyostelium* development at the mound stage (at 1.65 mM), whereas *dgkA^−^* cells were resistant to the compounds and developed into mature fruiting bodies (Fig. 5B). In a similar manner, the branched chain fatty acid 4-ethyloctanoic acid shows activity in both *Dictyostelium* phosphoinositide inhibition, in *ex vivo* hippocampal seizure models and in neuroprotection (Chang et al., 2012, 2013, 2015). This compound also blocked *Dictyostelium* development at the mound stage in wild-type cells (at 0.5 mM), but did not block the development of *dgkA^−^* cells (Fig. 5C). As a related control, a chemical with similar structure to these medium-chain fatty acids, 2-methylhexanoic acid, that showed no effect on *Dictyostelium* phosphoinositide regulation (Chang et al., 2012), and no effect in seizure control (Chang et al., 2013), was used. For this compound, both wild-type and *dgkA^−^* cells showed similar sensitivity during development (shown for 0.5 mM), with both cell types showing development arrest at the mound stage (Fig. 5D). These results suggest that a range of compounds, identified in *Dictyostelium* through the inhibition of phosphoinositide regulation and providing strong seizure control in mammalian models, have reduced potency in *dgkA^−^* cells, suggesting that the effect of these compounds is dependent upon the presence of DGKA.

We continued the analysis of *dgkA^−^* cells in response to the bipolar disorder treatment lithium. In *Dictyostelium*, lithium provides a well-described effect through inhibiting development related to a block in inositol phosphate signalling (Williams et al., 1999, 2002). To test for resistance to the developmental effect of lithium following loss of *dgkA*, development assays were repeated using lithium chloride (at 8 mM). In wild-type cells, development was blocked after aggregation, in early development, at first finger stage (Fig. 5E), as previously reported (Williams et al., 1999, 2002). In contrast, *dgkA^−^* cells were able to develop into mature fruiting bodies, showing distinct stalks and spore heads, following lithium treatment. These results suggest that the developmental effect of lithium chloride occurs through a mechanism dependent upon the presence of DGKA.

### Investigating a role for DGKA as a regulator of the effect of epilepsy and bipolar treatments on DAG levels

**Fig. 6.**
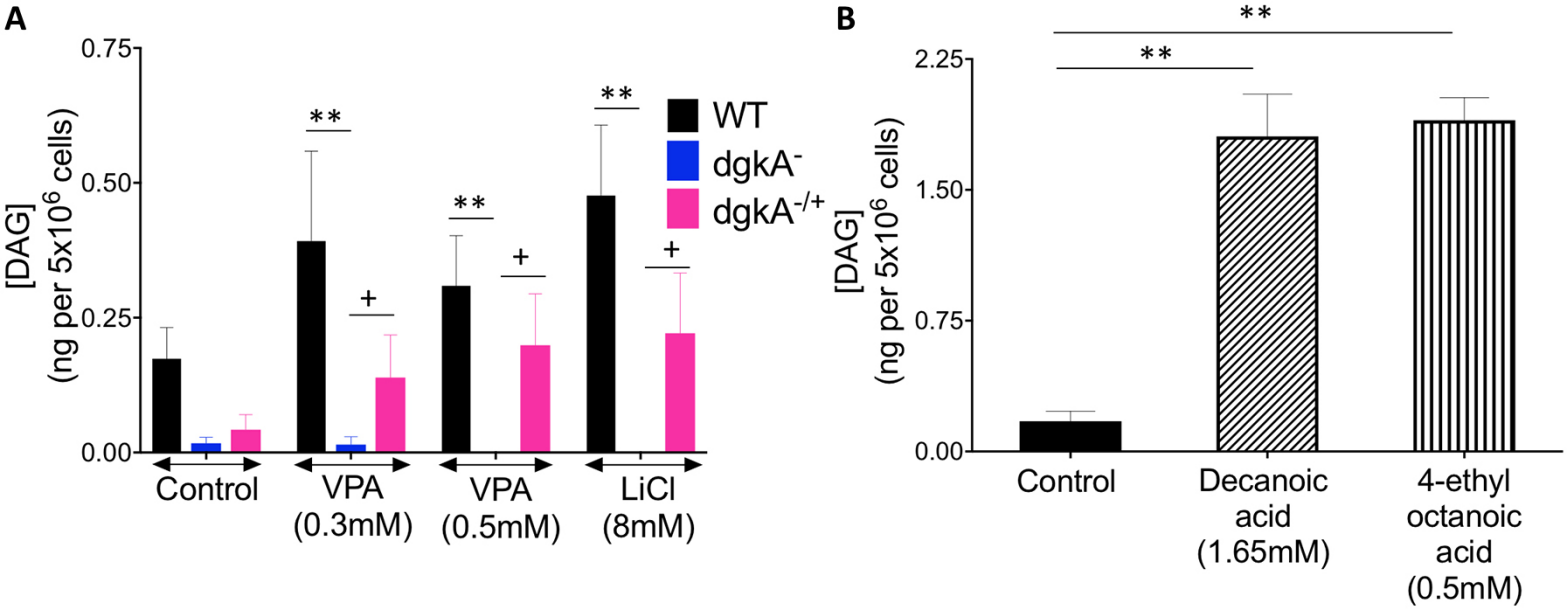
***Dictyostelium* DGKA-dependent DAG regulation following treatment with VPA, lithium and novel antiepileptic compounds.** WT, *dgkA^−^* and *dgkA^−/+^* cells were induced into early development by pulsatile cAMP and exposed to VPA, lithium or novel antiepileptic treatments, as indicated, for 10 min prior to ELISA quantification. (A) DAG levels in WT (black), *dgkA^−^* (blue) and *dgkA^−/+^* (pink) cells under control conditions and in the presence of 0.3 mM and 0.5 mM VPA and 8 mM LiCl. (B) DAG levels in WT cells in the absence (control) and presence of decanoic acid (1.65 mM) and 4-ethyloctanoic acid (0.5 mM). Data are mean±s.e.m. A Mann–Whitney test was used to compare the different cell lines and conditions, with a significant difference shown between WT and *dgkA^−^* cells (**P<0.01) and between *dgkA*^−^ and *dgkA^−/+^* cells (^+^*P*<0.05). Data are representative of quadruplicate independent experiments with duplicate technical repeats.

We then went on to quantify the molecular changes in *Dictyostelium* DAG levels in the presence and absence of DGKA, and following treatment with VPA, lithium and other compounds, using DAG-specific enzyme-linked immunosorbent assay (ELISA). In these experiments, cells were prepared in the same way as for acute cell behaviour experiments, with pulsatile cAMP to induce early development. Results from this approach surprisingly showed that, in the absence of treatment, *dgkA^−^* cells showed lower levels of DAG compared with wild-type cells, which were only partially restored on reintroduction of the enzyme within *dgkA^−/+^* cells (Fig. 6A). We then treated cells with VPA at concentrations in which *dgkA^−^* cells were resistant to treatment. In the presence of VPA (0.3 mM and 0.5 mM), DAG levels significantly increased in wild-type and *dgkA^−/+^* cells compared with *dgkA^−^* cells (Fig. 6A). In a similar fashion, cells treated with lithium chloride (8 mM) also showed significantly increased DAG levels. We also analysed the two unrelated structures with demonstrated seizure control activity, again at concentrations shown to block development in wild-type cells [decanoic acid (1.65 mM) and 4-ethyloctanoic acid (0.5 mM) (Chang et al., 2012, 2013, 2015)], and found that these compounds elevated DAG levels significantly above those in control (untreated wild-type) cells (Fig. 6B). These results suggest that, in *Dictyostelium*, loss of DGKA reduces DAG levels, and treatment of wild-type cells with VPA, lithium and new compounds, gives rise to a common effect of increasing DAG levels.

## DISCUSSION

Mechanistic insights into the biochemical basis of therapeutic treatments provides opportunities to increase our understanding of signalling pathways underlying these disorders and for the development of new treatments. In this study, we employed the simple biomedical model system *Dictyostelium* to investigate the cellular mechanisms of the epilepsy and bipolar disorder treatment, VPA, through the key enzyme, DGKA, which is responsible for the phosphorylation of DAG to PA. In mammalian models, DAG is a second messenger derived from phosphatidylinositol 4,5-bisphosphate (PIP_2_) through excitatory neurotransmitter activation of metabotropic glutamate receptors to activate PLC (Rodriguez de Turco et al., 2001). DAG has numerous roles including in the activation of PKCs (Newton, 1997), transient receptors channels (Lucas et al., 2003) and the endocannabinoid system (Williams et al., 2003). The roles of DGK thus include turning off DAG-dependent signalling, in addition to acting at the start of the phosphoinositide salvage pathway for the recycling of phosphoinositide signalling. By using *Dictyostelium* as a model, we were able to ablate all enzyme activity through loss of one protein, likely to represent the evolutionary origins of the family of mammalian DGK enzymes, with conserved structure and key amino acids. Consistent with the viability of yeast mutants, the *Dictyostelium* enzyme is not vital (Han et al., 2008a,b), although the encoded protein more closely resembles that found in higher eukaryotes rather than that in other single-cell models, such as yeast and bacteria (Cai et al., 2009). We show that loss of the *Dictyostelium dgkA* gene significantly reduced sensitivity to the acute effects of VPA on changes in cell behaviour and in multicellular development, suggesting that the effect of VPA on cellular function is at least partially dependent upon DGKA in *Dictyostelium*. It is therefore likely that VPA acts to change a cellular function in this model to cause acute and developmental effects, where these changes are dependent on the presence of DGKA, thus implicating either perturbed DGKA-related signalling or DGKA activity as a cellular mechanism for VPA action. We further used *Dictyostelium* development to analyse a range of compounds associated with both epilepsy and bipolar disorder treatments, to show that loss of DGKA decreases sensitivity to both treatments. At a biochemical level, loss of DGKA appears to reduce DAG levels, and exposure to both epilepsy and bipolar disorder treatments increases DAG levels. Removal of DGKA therefore counteracts the effect of these treatments in increasing DAG levels. These studies thus suggest that, in *Dictyostelium*, DGKA might regulate a cellular mechanism common for drugs used to treat both epilepsy and bipolar disorder.

In this study, we used the *dgkA^−^* cell line to assess a role for DGKA in the biochemical activity of VPA. This approach is unusual, as we have removed all DGK activity in these cells. Our earlier studies have shown that VPA acutely blocks cell behaviour, with a concomitant reduction in phosphoinositide levels (Chang et al., 2012; Xu et al., 2007), and reduces inositol phosphate levels (Williams et al., 1999). Here, we extend these studies, using identical conditions, to show that loss of DGKA reduces the acute effect of VPA on cell behaviour, and blocks the VPA-dependent increase in DAG levels in wild-type cells. The most likely rationale for this resistant phenotype is that of molecular changes in the *dgkA^−^* mutant, either through loss of the enzyme as a direct target for VPA or by alterations in cell signalling regulated by DGKA to overcome the cellular effect of VPA in this model. Further studies are necessary to investigate a direct role for VPA in inhibiting DGKA, and to examine the VPA-dependent regulation of the phosphoinositide salvage pathway (Fakas et al., 2011; Rodriguez de Turco et al., 2001; Walsh et al., 1995), phosphoinositide levels (Chang et al., 2012; Xu et al., 2007) and inositol phosphates (Williams et al., 1999, 2002).

This study provides the first analysis of *Dictyostelium* DAG levels. We show that nanomolar levels of DAG in unstimulated *Dictyostelium* cells (at ∼0.4 ng/10^7^ cells) are similar to those reported for human tissue (110 nmol/g), pigs (using porcine aortic endothelia cells) (13.7 nmol/10^7^ cells) and mice fibroblast Swiss 3T3 cells (1.4 nmol/10^7^ cells) (Pettitt et al., 1997; Szendroedi et al., 2014). Although *Dictyostelium* DAG levels were shown to be variable, consistent with those shown in *in vivo* mouse model studies (Rodriguez de Turco et al., 2001), the *dgkA^−^* mutant showed a significant and large reduction in DAG levels. This result was unexpected, because elevated levels of DAG would be expected following removal of DGKA. Two potential mechanisms can be suggested for this result. First, the cellular response VPA treatment (or *dgkA* deletion) might be to upregulate the activity of the Kennedy pathway, through which DAG is recycled to PA through PE and PC to form CDP-DAG (Gibellini and Smith, 2010). This theory appears likely, because VPA treatment in *Dictyostelium* leads to the accumulation of nonpolar lipids in lipid droplets typically consisting of DAG and triacylglycerol (TAG) species (Kalantari et al., 2010), and includes PE and PC (Elphick et al., 2012)*.* Thus, enhanced activation of the Kennedy pathway might be induced by acute exposure to VPA or to a chronic block in DGKA activity (through gene ablation). A second potential mechanism could simply involve the stabilisation or protection of DAG levels through binding to DGKA, thus loss of the protein (as distinct to inhibition of the enzyme) leads to the reduction in DAG levels. It must also be acknowledged that VPA-dependent changes in DAG, although providing a useful marker, could indeed arise through effects on a variety of other metabolites to give rise to this effect. However, through either direct or indirect processes, treated *Dictyostelium* cells showed a significantly altered DAG level, suggesting that DAG-dependent signalling might provide an important target pathway for VPA, lithium and other compounds.

DGK-related activity might play an important role in epilepsy. A range of studies have demonstrated a role for phosphoinositide signalling in both seizure susceptibility and progression (Backman et al., 2001; Chang et al., 2014; Shinoda et al., 2004; Vanhaesebroeck et al., 2012), and this signalling is dependent upon DAG recycling through DGK activity. In addition, mutations in several DGK isoforms have been linked with increased seizure activity (Ishisaka et al., 2103; Leach et al., 2007; Rodriguez de Turco et al., 2001). The data provided here suggest that, in *Dictyostelium*, one action of VPA is related to the cellular function of DGK, because ablation of DGKA attenuates the effect of VPA. Further studies will be necessary to investigate a VPA-dependent effect on seizure control through regulation of DGK-related signalling. It is also interesting to note that previous studies have shown a reduction in dendritic spines in epilepsy patients (Muller et al., 1993; Scheibel et al., 1974), that DGKβ controls dendritic spine outgrowth and maturation (Hozumi et al., 2009), and that VPA treatment increases dendritic spine formation in mouse models (Yang et al., 2016). Together, these data provide a view consistent with a role for VPA in regulating cell function through alterations in DGK-dependent signalling pathways related to seizure activity and neurological effects.

We also sought to investigate a role for *Dictyostelium* DGKA in attenuating the effects of other potential epilepsy treatments. Here, we employed a range of novel compounds, identified in *Dictyostelium* to provide a similar cellular function to VPA, that show seizure control effects in mammalian models (Chang et al., 2016, 2012, 2013, 2014, 2015). Importantly, these compounds lack the potential adverse effect of histone deacetylase inhibition at therapeutically relevant concentrations, and this effect has been linked to teratogenicity of VPA and resulting birth defects (Jentink et al., 2010). Using the *Dictyostelium* development assay to screen compounds, we showed that loss of DGKA also provided reduced sensitivity to decanoic acid [the therapeutically active constituent of the medium-chain-triglyceride ketogenic diet (Augustin et al., 2018)] and to 4-ethyloctanoic acid. In contrast, 2-methyhexanoic acid, a related compound with low activity in *Dictyostelium* without seizure control activity (Chang et al., 2012, 2013), did not show differential effects between the two cell lines. Furthermore, decanoic acid and 4-ethyloctanoic acid treatment also elevated DAG levels at concentrations shown to block development in wild-type cells, although the large increase in DAG levels shown here should be investigated further to exclude potential nonspecific effects on the assay. These studies therefore propose a role for DGKA in regulating the cellular effects of other potential epilepsy treatments, beyond that of VPA.

DGK-regulated signalling also provides an attractive therapeutic target for bipolar disorder. Data provided here demonstrate that the previously reported effect of the bipolar disorder treatment lithium on *Dictyostelium* development is overcome by loss of DGKA (Boeckeler et al., 2006; Williams et al., 2002). Earlier studies in *Dictyostelium* have shown that both VPA and lithium attenuate inositol phosphate signalling as a mechanism for bipolar disorder treatments (Eickholt et al., 2005; Shimshoni et al., 2007; Williams et al., 2002, 1999). The reduction in phosphoinositide signalling caused by VPA, through attenuation of DGKA, might therefore underlie this change in inositol phosphate levels as a potential mechanism for bipolar disorder treatment. This association is further supported by the role of DAG in bipolar disorder-associated signalling. In mammalian models, DGK competes with PKC for binding to DAG, thereby regulating PKC activity, and dysregulation of the PKC signalling pathway has been widely demonstrated in bipolar disorder patient populations and following therapeutic treatment (Sakai and Sakane, 2012; Saxena et al., 2017). Numerous studies have identified upregulated PKC activity in bipolar disorder patients (Friedman et al., 1993; Wang and Friedman, 1996), with this activity being reduced by both lithium (Friedman et al., 1993; Wang and Friedman, 1989) and VPA (Ramadan et al., 2011; Watson et al., 1998) in animal models and patient studies (Katsel et al., 2008; Soares and Mallinger, 1997; Wang et al., 2001). Associations with bipolar disorder have been made with DGKβ and DGKη (Baum et al., 2007; Kakefuda et al., 2010; Moya et al., 2010; Squassina et al., 2009), with lithium treatment resulting in accumulation of DAG in a range of models (Brami et al., 1993; Drummond and Raeburn, 1984), independent of phospholipase D (Nilssen et al., 2005). DGK also interacts with NR5A1, which in turn binds to phosphatidylinositol headgroups, in particular PI(3,4)P_2_ and PI(3,4,5)P_3_, providing a mechanism of DGK-dependent regulation of phosphoinositides beyond that of DAG recycling (Blind et al., 2014). Thus a VPA- and lithium-dependent effect on signalling components controlled by, or involving, DGK activity could provide a biochemical mechanism underlying the treatment of patients with bipolar disorder.

Developing new treatments for bipolar disorder has remained a difficult prospect owing to a lack of clarity regarding a therapeutic mechanism of current drugs. One approach to overcome this is based upon the observation that a range of antiepileptic drugs are also effective in the treatment of bipolar disorder (Rogawski and Loscher, 2004). In earlier studies, we have successfully translated a common molecular mechanism relating to both epilepsy and bipolar disorder treatments from *Dictyostelium* to mammalian neurons, demonstrating that it is possible to model bipolar disorder drug mechanisms in *Dictyostelium* (Williams et al., 2002, 1999). In our current study, we show that novel compounds (decanoic acid and 4-ethyloctanoic acid) function through modulating a DGKA-related pathway commonly affected by lithium and VPA, suggesting that these compounds should be investigated as new treatments for bipolar disorder as well as epilepsy.

**Fig. 7.**
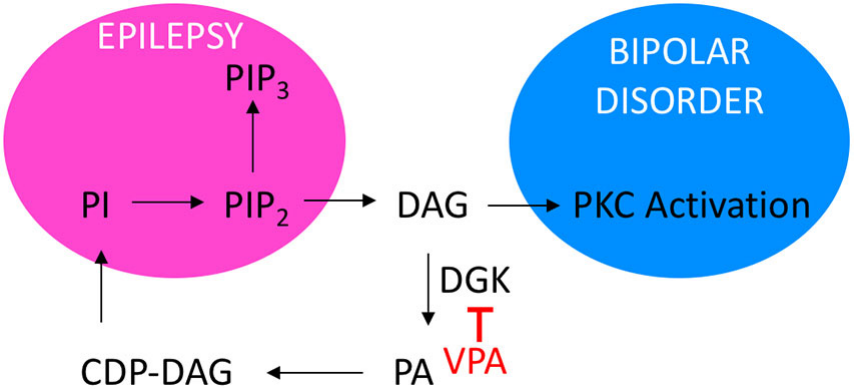
**Proposing a mechanism for VPA, linking epilepsy and bipolar disorder with the phosphoinositide salvage pathway.** Phosphoinositide signalling (pink oval) has been widely shown to be deregulated in seizures and epilepsy, is regulated by VPA, and is dependent upon the salvage pathway involving DAG and DGKs. In addition, protein kinase C (PKC) activity is regulated by DAG, and numerous studies have described changes in PKC pathway activation in bipolar disorder studies (blue oval), in which patients are treated with VPA or lithium. Our data, based on the model system *Dictyostelium*, suggest that loss of the DGK enzyme reduces the effect of both VPA and lithium, and reduces DAG levels, and that both compounds (and other potential new epilepsy treatments) function to commonly elevate DAG levels. Further pre-clinical and clinical studies are therefore needed to investigate DAG and related signalling as a target pathway in the treatment of both disorders.

In summary, VPA is a widely used treatment for both epilepsy and bipolar disorder, without a clear mechanism of action and with significant side effects. Here, we propose that signalling regulated by DGK could provide a key role in the mechanisms of action for VPA (Fig. 7), in addition to other proposed therapeutic compounds that could provide treatments for both epilepsy and bipolar disorder.

## MATERIALS AND METHODS

### Reagents

All compounds were purchased from Sigma-Aldrich (Dorset, UK) unless otherwise stated. Axenic medium, SM agar, phosphate buffer (KK2) and blasticidin were obtained from ForMedium (Norfolk, UK). Penicillin-streptomycin was purchased from Gibco (Paisley, UK), decanoic acid was from Alfa Aesar (Massachusetts, USA) and all enzymes were purchased from Thermo Fisher Scientific (Hemel Hempstead, UK).

### Cell culture, strains and plasmids

*Dictyostelium* cell lines were grown at 22°C in Axenic medium which contained 100 µg/ml penicillin-streptomycin. *dgkA^−^* cell lines were generated from wild-type (Ax2) cells. Creation of the *dgkA* knockout construct was as previously described (Pakes et al., 2012). DNA was amplified from the 5′ and 3′ regions of the *dgkA* gene (DDB_G0277223) (Fey et al., 2013) and cloned into the pLPBLP vector on either side of a blasticidin resistance cassette using the restriction enzymes *BamH*I, *Pst*I, *Nco*I and *Hind*III. The knockout cassette was excised from the vector through restriction digestion and electroporated into wild-type cells. Transformants were selected in medium containing 10 µg/ml blasticidin, and surviving colonies were screened by PCR for homologous integration using a genomic and vector control bands and a diagnostic band. RT-PCR (First Strand cDNA Synthesis Kit, Thermo Fisher Scientific) was performed to confirm loss of gene transcription. The *dgkA^−/+^* rescue cell line was produced using the pTX-FLAG-DGK-GFP plasmid vector, which was kindly provided by T. Egelhoff (De La Roche et al., 2002).

### *Dictyostelium* random cell movement assay

As previously described (Cocorocchio et al., 2016, 2018), wild-type and transformant cells (1×10^7^) were harvested from mid-log-phase-growing shaking cultures and washed in phosphate buffer, resuspended at 1×10^7^ cells in 6 ml phosphate buffer, and pulsed with 30 nM cAMP at 6 min intervals for 5 h at 120 rpm. Cells were further washed in phosphate buffer, resuspended in 4 ml phosphate buffer and diluted 1:10. Cells (250 µl) were placed in a Nunc Lab-Tek coverglass chamber (Thermo Fisher Scientific) and incubated for 15 min in order for the cells to adhere prior to time-lapse microscopy for 900 s. After a control period of 225 s, 250 µl of double concentrate compound was added, and change in cell behaviour was monitored at 30 cells per condition from at least three independent experiments. To analyse random cell movement, the ImageJ Quimp 11b software package and accompanying scripts for MATLAB analysis were used. Individual cells were segmented and cell behaviour before and after drug addition were quantified using measures of circularity (where the ratio of perpendicular axes through each cell provides a ratio, with ‘1’ indicating a perfect circle), protrusion formation (defined within motility maps as regional peaks exceeding an average speed of 0.1 µm/s, and counted automatically over a running window of 10 frames) to represent protrusive activity within short time periods, centred around discrete time points) and motility between each frame (local membrane velocities between frame motility maps), counted automatically over a ten-frame running window.

### *Dictyostelium* development assay

*Dictyostelium* wild-type and mutant cells (1×10^7^ cells) were washed in phosphate buffer and developed on a nitrocellulose membrane filter above a hydrophobic membrane filter (both from Millipore, Watford, UK) in the absence or presence of compounds. Filters were incubated for 24 h at 22°C prior to imaging. Experiments were repeated over at least three independent experiments.

### DAG analysis

Levels of DAG were determined using ELISA (General ELISA Kit for Diacylglycerol, E2038Ge, Amsbio, Abingdon, UK). Cells were prepared by pulsing with cAMP (as described for cell behaviour assays). DAG levels were then measured from 5×10^6^ cells, treated with VPA (0.3 mM–0.5 mM) or lithium chloride (8 mM) for 10 min in shaking suspension. Cells were washed in phosphate buffer and resuspended in 100 µl phosphate buffer. The cell wall fraction was obtained from six rounds of freeze-thaw (3 min each), centrifuged (10,000 ***g***, 10 min, 4°C) and the pellet was resuspended in 100 µl sample diluent from the kit. ELISA was conducted following the supplier's instructions. Data are derived from at least triplicate independent experiments.

### Data analysis and statistics

In the analysis of cell behaviour, a Kruskal–Wallis analysis with Dunn’s post hoc test was employed to test for statistically significant changes between three independent groups (wild type, *dgkA^−^* and *dgkA^−/+^*) that were not normally distributed. Differences were considered statistically significant if the *P*-value was less than 0.05. In the analysis of DAG levels, a Mann–Whitney analysis was employed to test for statistically significant changes between groups, again with data that were not normally distributed but enabling direct paired groups.

## Supplementary Material

Supplementary information

First Person interview
