# Supplementary material for: Diacylglycerol kinase (DGKA) regulates the effect of the epilepsy and bipolar disorder treatment valproic acid in Dictyostelium discoideum
Source: Dis Model Mech. 2018 Aug 21;11(9):dmm035600. doi: 10.1242/dmm.035600 (PMC6176992; doi:10.1242/dmm.035600)
Supplement: Supplementary information [file dmm-11-035600-s1.pdf]

FIRST PERSON

First person – Elizabeth Kelly

First Person is a series of interviews with the first authors of a selection of papers published in Disease Models & Mechanisms, helping early-career researchers promote themselves alongside their papers. Elizabeth Kelly is first author on 'Diacylglycerol kinase (DGKA) regulates the effect of the epilepsy and bipolar disorder treatment valproic acid in *Dictyostelium discoideum*', published in DMM. Elizabeth is a PhD student in the lab of Robin Williams at the Royal Holloway University of London, Surrey, UK, where her research focuses on neurology, drug discovery and immunology.

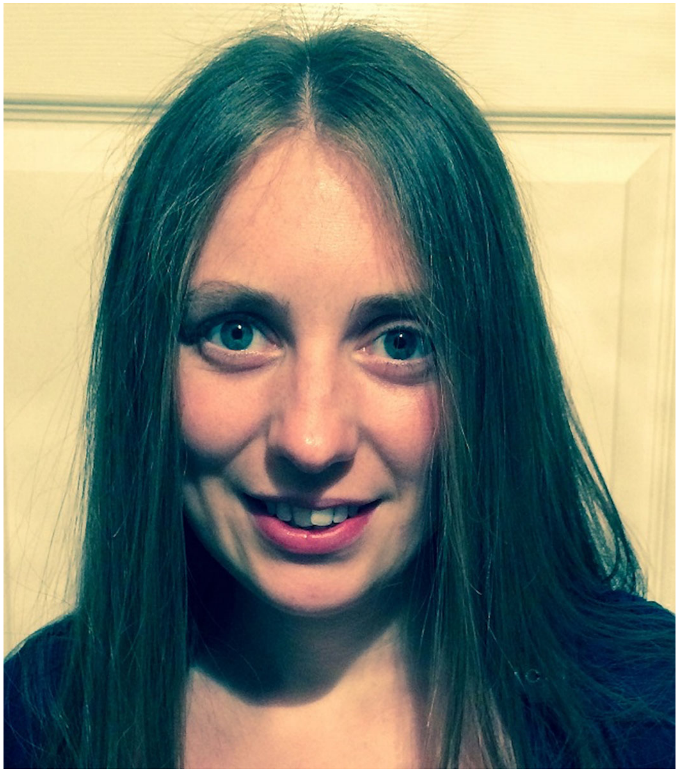

Elizabeth Kelly

How would you explain the main findings of your paper to non-scientific family and friends?

Alternative epilepsy treatments are required for patients who continue to experience seizures. One of the most successful epilepsy treatments is the drug valproic acid (VPA). The purpose of this study is to identify how VPA prevents seizures in epilepsy. This was achieved by investigating individual targets within a biological pathway. The long-term goal is to develop new anti-epileptic drugs that target the same enzyme but do not cause defects in developing embryos. The best way to describe this is using cars on a motorway. Think about the ordinary system of a motorway, with a number of lanes and cars leaving and going onto a junction. If the drug blocks two lanes of a motorway, a traffic jam will build up. This study has focused on using the amoeba *Dictyostelium discoideum* as a model to discover what VPA regulates to cause the traffic jam. It was found that a range of anti-seizure compounds, including VPA and the bipolar disorder drug lithium chloride, regulate a *D. discoideum* enzyme called DGKA, and that in the absence of DGKA, treatment with these compounds results in no traffic jam, allowing cars to move freely. The results emphasize a link between epilepsy and bipolar disorder, like the junctions on a motorway. Combining the results of this study with previously published literature, it can be hypothesised that deletion of DGKA, or its regulation with anti-seizure drugs like VPA and lithium chloride, causes a traffic jam where the resulting traffic then leaves the motorway at a junction, follows diversion signs to bypass the blockage along an alternative pathway, and then re-joins the motorway at another junction.

"These results could considerably benefit the group of epilepsy and bipolar disorder patients resistant to currently available medication"

What are the potential implications of these results for your field of research?

From the results presented in this paper, it seems that the mechanism of action of VPA involves regulating the single diacylglycerol kinase (DGK) isoform found in *D. discoideum*, DGKA. Therefore, new anti-epileptic treatments that lack the teratogenic properties of VPA can be developed around DGKs, such as the anti-seizure compounds decanoic acid and octanoic acid. The results from this paper also show that lithium chloride has a similar mechanism of action as VPA, through regulating DGKA. As both VPA and lithium chloride cause an increase in diacylglycerol levels, which was abolished in the knockout mutant, regulation of DGK appears to link epilepsy and bipolar disorder. These results could considerably benefit the group of epilepsy and bipolar disorder patients resistant to currently available medication; if the mechanism of both disorders involves regulating DGK, then treatments for either disorder could potentially be used to treat the other. In addition, new epilepsy and bipolar disorder treatments can be developed around regulating DGK.

What are the main advantages and drawbacks of the model system you have used as it relates to the disease you are investigating?

The advantages of using *D. discoideum* as a model in this study is that the amoeba has only one DGK isoform, compared with mammalian models that have ten. In addition, VPA has previously been shown to have an effect on *D. discoideum* cell growth, acute cell behaviour and development, enabling drug-resistant cell lines to be visualised. As VPA and lithium chloride were seen to regulate DGKA activity in *D. discoideum*, this work will need to be replicated in mammalian epilepsy models.

Elizabeth Kelly's contact details: Centre of Biomedical Sciences, School of Biological Sciences, Royal Holloway University of London, Egham, Surrey TW20 0EX, UK.
E-mail: Lizzie.Kelly.2007@live.rhul.ac.uk

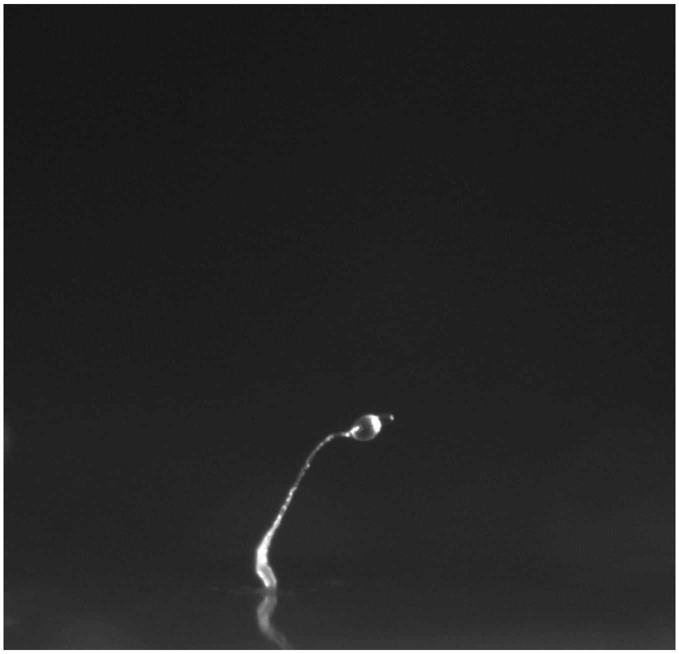

Single *dgkA* null fruiting body showing resistance to 0.5 mM VPA.

Describe what you think is the most significant challenge impacting your research at this time and how will this be addressed over the next 10 years?

The most significant challenge of this work is that VPA has previously been found to regulate a number of pathways, including inhibiting voltage-gated calcium, potassium and sodium channels, histone deacetylase and *N*-methyl-D-aspartate excitation; enhancing gamma-amino butyrate; and reducing inositol phosphate and phosphoinositide levels. This makes identifying the target of VPA challenging. The work in this study has, therefore, focused on identify the mode of action of VPA in relation to reducing phosphoinositide levels, which was found to be through regulating DGK. If new anti-epileptic drugs can be developed that target DGK and have a similar potency to VPA but lack the side effects of hepatoxicity and teratogenicity in the next ten years, there would be a considerable benefit for individuals with epilepsy and bipolar disorder.

What's next for you?

Once I have completed my PhD I hope to obtain a research position either as a postdoc or in a large multinational pharmaceutical company.

Reference

Kelly, E., Sharma, D., Wilkinson, C. J. and Williams, R. S. B. (2018). Diacylglycerol kinase (DGKA) regulates the effect of the epilepsy and bipolar disorder treatment valproic acid in *Dictyostelium discoideum*. *Dis. Model. Mech.* **11**: dmm035600.
